# Homœopathy and Rational Medicine

**Published:** 1901-02-09

**Authors:** R. E. Dudgeon

**Affiliations:** 63 Upper Berkeley Street, W.


### Editor's Letter-Box.

Our Correspondents are reminded that prolixity is a great bar to publication, and that brevity of style and conciseness of statement greatly facilitate early insertion.]

### HOMŒOPATHY AND RATIONAL MEDICINE.

To the Editor of *The Hospital*.

Sir,—I have only to-day seen your notice, in the No. for January 12th, of my article "A Century of Homœopathy.' If it be granted that yours is "rational medicine," your critical remarks are justified. But does the ordinary practice of the dominant school represent rational medicine? The only reason you give for the selection of a remedy for a disease is that you want to be satisfied "whether it really does good." It is a matter of complete indifference to you "whether the beneficial effect of a certain drug is discovered by a homœopathist, an old woman, or a naked savage." You don't care a straw *why* the drug cures, you only take the word of the naked savage, the old woman, or the homœopathist that it does cure as your reason for employing it. Is that rational medicine? I would call it pure empiricism, or eclecticism, if you prefer the term. The homœopathist sets to work to find remedies for cases of disease in a different way. We first ascertain the effects of drugs by testing them on the healthy, and, in any case of disease, we give a medicine that causes on the healthy an array of symptoms as like as possible to those of the disease; in other words, we give a remedy which we have found to act on precisely the same parts as are affected in the disease to be cured.

You do not deny that homœopathists discover remedies by their "provings," and you say, "let us give them every credit for it." We are grateful for this admission. Had it been usual in your school we should not have talked of you pillaging our materia medica, for you are heartily welcome to all the fruits of our labours, if only you will have the courtesy and scientific spirit to acknowledge their source.

As to which side the *rapprochement* of the two systems comes from, I would only remind you that, since the introduction of homœopathy, your school has abandoned most of its cherished traditional methods of treatment, and now uses many of the remedies introduced to medicine by the homœopathists; whereas homœopathy has not changed its therapeutic rule since Hahnemann propounded it more than a century ago, and has not adopted any of the methods, either old or new, of your school. So that the *rapprochement* of the two practices can hardly be said to have come from our side.

Yours faithfully,

R. E. DUDGEON, M.D.

63 Upper Berkeley Street, W.,
February 2nd, 1901.

[We never either said or suggested that we "take the word of the naked savage, the old woman, or the homœopathist that it (that is the remedy) does cure," so that what Dr. Dudgeon says about our practice being "pure empiricism" falls to the ground. If Dr. Dudgeon will kindly refer to our article he will find that our remark about the "naked savage" was made to enforce the fact that the practitioners of what we have spoken of as "rational medicine" are in no way restricted in their choice of remedies, and also that we went on to say that what would influence us in the using of a drug would be "the accumulated experience of its beneficial action in certain morbid states and of its power to produce certain effects which we desire for the relief of our patients." We certainly never suggested that we would "take the word" of either the naked savage or the homœopathist on the question. As to the question of "*rapprochement*," we cannot see that the fact—if it is a fact—that our "school" (we quote the word although we do not approve of it) "has abandoned most of its cherished traditional methods of treatment and now uses many of the remedies introduced by homœopathists" gives the slightest excuse for suggesting any leaning towards homœopathy. Surely knowledge has advanced during the past hundred years, and as the very essence of rational medicine is that it brings all knowledge to bear on treatment, the practice of medicine must of necessity have advanced; "whereas," as Dr. Dudgeon says, "homœopathy has not changed its therapeutic rule since Hahnemann propounded it more than a century ago." If Dr. Dudgeon considers that our use of many of the remedies introduced to medicine by homœopathists indicates a leaning to homœopathy, or is, in his own words, "a sort of homœopathy," we must leave him to the tender mercies of his fellow-homœopathists. That is not our idea of homœopathy, nor, as we think, would it have been Hahnemann's.—Ed. The Hospital.]

